# Relationship between ghrelin and thyroid disease: a meta-analysis

**DOI:** 10.3389/fendo.2025.1505085

**Published:** 2025-02-28

**Authors:** Caihong Xin, Jiayi Yao, Huijuan Li, Xin Sun, Huijuan Wang

**Affiliations:** ^1^ Department of Endocrinology and Metabolism, Fourth People’s Hospital of Shenyang, Shenyang, China; ^2^ Department of Endocrinology and Metabolism, First Affiliated Hospital of Soochow University, Suzhou, China; ^3^ Department of Diabetes, Taiping Street Health Center of Xiangcheng District of Suzhou City, Suzhou, China

**Keywords:** ghrelin, thyroid disease, hyperthyroidism, hypothyroidism, meta - analysis

## Abstract

**Background:**

Previous studies have identified a close relationship between ghrelin levels and thyroid disease. Ghrelin levels were lower in patients with hyperthyroidism compared with healthy individuals, and increased after treatment. However, other studies have reported inconsistent results. As such, the association between ghrelin and thyroid disease remains controversial.

**Methods:**

A literature search of the Web of Science, Wiley Online Library, Embase, and PubMed databases was performed. The title or abstract search term “thyroid” was used in combination with “ghrelin”. Meta-analysis results are reported as standardized mean difference with corresponding 95% confidence interval (CI).

**Results:**

Twenty-three studies were included in this meta-analysis. Ghrelin levels in patients with hyperthyroidism were significantly lower than those in healthy individuals (SMD: -1.03, 95% CI [-1.75, 0.32]), but significantly higher after effective treatment (SMD: 0.77, 95% CI [0.03, 1.51]). Ghrelin levels were higher, but not significantly, in patients with hypothyroidism compared with healthy controls (SMD: 0.48, 95% CI [-0.13, 1.08]).

**Conclusions:**

This systematic review is the first to evaluate the relationship between ghrelin and thyroid disease. Determining the role of ghrelin in thyroid disease will significantly contribute to understand of symptom or pathomechanism.

**Systematic review registration:**

https://www.crd.york.ac.uk/prospero/, identifier CRD42024591501.

## Introduction

The thyroid is among the most important endocrine glands in the human body. Its main role is to synthesize and secrete thyroid hormones, which impact growth, metabolism, and development. Thyroid hormones can affect the nervous, circulatory, digestive, reproductive, and other systems, and play a role in regulating human energy metabolism. Thyroid dysfunction is the most common thyroid disease (1). Searching for mutual relationships between other hormones and the thyroid hormones is important for a better understanding of symptom or pathomechanism.

Ghrelin is an endogenous polypeptide composed of 28 amino acids, first identified in the gastric mucosa of rats by Kojima et al. in 1999. Ghrelin is the endogenous ligand of the growth hormone secretagogue receptor (GHS-R). Ghrelin has the highest concentration in the stomach and is expressed in the intestine, hypothalamus, pituitary, thyroid, heart, and other tissues, laying the foundation for its wide range of physiological effects. Ghrelin promotes the release of growth hormone, enhances appetite, regulates fat metabolism, protects the vascular endothelium, and improves cardiac function (2). Studies have shown that ghrelin can alter the morphology of thyroid-stimulating hormone-secreting cells and decrease serum thyroid-stimulating hormone levels when injected into the lateral cerebral ventricles of rats (3). Patients with thyroid dysfunction exhibit a variety of abnormalities, such as changes in thyroid hormone levels and autoimmune disorders, which are often accompanied by abnormal energy metabolism of glucose and lipids. In recent years, changes in ghrelin levels in this state have gradually become a “hot topic” of research. Riis et al. found that ghrelin levels were significantly lower in patients with hyperthyroidism than those in healthy individuals, and were significantly increased after treatment (4). Gjedde et al. reported a significant increase in ghrelin levels of patients with hypothyroidism than healthy controls and ghrelin levels was significant decreased after treatment of hypothyroidism (5). However, the results of other similar studies were inconsistent with those of earlier investigations (6, 7). The ghrelin levels of hyperthyroidism and hypothyroidism were not significant change in some studies. Therefore, the relationship between ghrelin levels and thyroid disease remains controversial. Whether changes in ghrelin levels are related to thyroid disease remains to be confirmed. As such, the present meta-analysis aimed to systematically and comprehensively evaluate the relationship between ghrelin levels and thyroid disease.

## Methods

### Literature search

A literature search of the Web of Science, Wiley Online Library, PubMed, and Embase databases was performed. The search scope included studies investigating the relationship between ghrelin and patients with thyroid disease, published up to October 2024. The title or abstract search terms “ghrelin” was used in combination with “thyroid”. Eligible studies were restricted to those published in English. The reference lists of the retrieved studies were manually searched to identify additional, potentially eligible studies. The present study was registered in the PROSPERO Database (CRD42024591501). All requisite items reported for systematic reviews and meta-analyses are listed in the **Supplementary Table S1**.

### Inclusion criteria

Meta-analysis was performed on studies fulfilling the following criteria: sufficient data regarding ghrelin levels in patients with thyroid disease and healthy controls; case-control or cohort design; and publication language in English.

### Exclusion criteria

Duplicate studies, those with insufficient data, samples without serum and plasma data, animal studies, meta-analyses, reviews, meeting summaries, case reports, and editorials were excluded.

### Data extraction

Two investigators independently reviewed the titles and abstracts of the retrieved studies according to predetermined inclusion and exclusion criteria. Disagreements regarding study selection during the review process were resolved through consensus discussion based on an established standard. If consensus could not be reached, a third investigator was invited to participate in the final decision as to whether the study fulfilled the inclusion criteria. Data extracted from the selected studies included the following: first author; publication year; region; design; sample; and disease.

### Risk of bias

The Cochrane Collaboration recommends the Newcastle-Ottawa Scale (NOS) as a tool for assessing bias in observational studies (8). The NOS was used as an evaluation standard, and two researchers were based on the research content of the case-control studies and cohort studies, the methodological quality of independent evaluation, including selection of the research objective, comparability between groups, and outcome measures. An NOS score between 0 and 9 was calculated, with ≥ 6 points considered to be a high-quality study; the higher the score, the higher the quality of the study.

Bias was also assessed with the ROBINS-I (risk of bias in non-randomized studies-of interventions) tool for nonrandomized studies. The ROBINS-I tool views each study as an attempt to emulate a hypothetical pragmatic randomized trial and assesses seven domains through which bias might be introduced: bias due to confounding, bias in the selection of participants into the study, bias in classification of interventions, bias due to deviations from intended interventions, bias due to missing data, bias in the measurement of outcomes, and bias in the selection of the reported result. The judgments within each domain carry forward to an overall risk of bias of: ‘Low’, ‘Moderate’, ‘Serious’, ‘Critical’ or ‘No information’ (9).

### Statistical analysis

Results are expressed as standardized mean difference (SMD) with corresponding 95% confidence interval (CI). Heterogeneity was assessed according to I^2^ and *P* values, as follows: I^2^ < 50%, *P* > 0.1 represented low heterogeneity, and a fixed effects model was used for analysis; I^2^ ≥ 50% and *P* ≤ 0.1 represented high heterogeneity, and a random effects model was used for analysis. Sensitivity analyses were performed on the sources of heterogeneity in the outcome indicators. Egger’s test was used to assess publication bias. Differences with *P* < 0.05 were considered to be statistically significant. Statistical analysis was performed using Stata Release 12.0 (StataCorp LLC, College Station, TX, USA).

## Results

### Literature search and study selection

**Figure 1:**
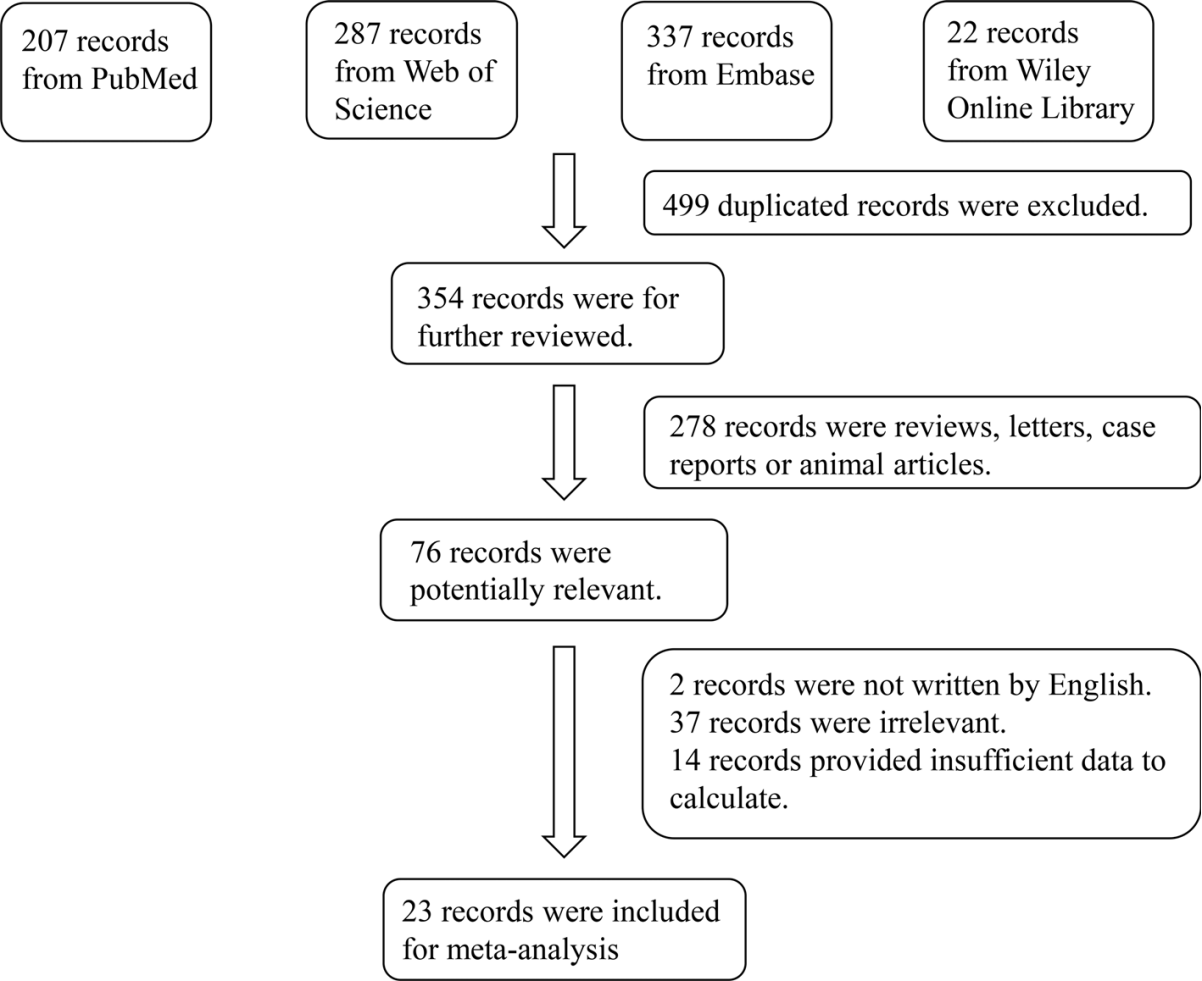
Flowchart of the detailed procedure for the inclusion or exclusion of selected studies.

A total of 853 relevant studies were retrieved from the Web of Science, Wiley Online Library, PubMed, and Embase databases, of which 499 duplicates were excluded, and 278 were excluded after reading titles and abstracts. An additional 53 studies were excluded after reading the full texts; ultimately, therefore, 23 studies comprising 1089 patients with thyroid disease and 693 controls were included in the meta-analysis (4–7, 10–28). A flow diagram illustrating the study selection process is presented in **Figure 1**. The characteristics of the included studies are summarized in **Table 1**. All 23 studies included in this meta-analysis fulfilled the criteria for NOS categories of selection, comparability, and exposure. The ROBINS-I assessment of study bias for included studies was presented in **Supplementary Table S2**.

**Table 1:** Study characteristics of the published studies included in the meta-analysis.

Author	Publication Year	Region	Design	Number		Disease	Sample
				Case	Control		
Riis ALD (4)	2003	Denmark	Case-control and cohort study	9	8	Hyperthyroidism (Graves’ disease)	Serum
Rojdmark S (10)	2005	Sweden	Case-control and cohort study	11	16	Hyperthyroidism (Graves’ disease)	Serum
Morpurgo PS (11)	2005	Italy	Case-control study	16	15	Thyroid carcinoma (medullary thyroid carcinomas)	Serum
Gimenez-Palop O (12)	2005	Spain	Case-control and cohort study	41	45	The hyperthyroid group consisted of 24 patients, and etiology of the hyperthyroidism was Graves’ disease (n = 23) and toxic multinodular goiter (n = 1). The hypothyroid group consisted of 17 women and the etiology of the hypothyroidism was chronic autoimmune thyroiditis (n = 6), radioiodine therapy (n = 6), withdrawal of thyroid hormone therapy before a total body scan for papillary thyroid carcinoma (n = 3), non-autoimmune hypothyroidism (n = 1) and postpartum thyroiditis (n = 1).	Plasma
Altinova AE(a) (13)	2006	Turkey	Case-control study	48	43	Hyperthyroidism (Graves’ disease)	Serum
Altinova AE(b) (7)	2006	Turkey	Case-control and cohort study	47	48	Hypothyroidism (Hashimoto’s thyroiditis)	Serum
Gjedde S (5)	2008	Denmark	Case-control and cohort study	11	10	Hypothyroidism (Hashimoto’s thyroiditis)	Serum
Br Clik M (14)	2008	Poland	Case-control and cohort study	28	19	The hyperthyroid group consisted of 17 patients, and etiology of the hyperthyroidism was Graves’ disease (n = 10) and toxic multinodular goiter (n = 7). The hypothyroid group consisted of 11 patients and the etiology of the hypothyroidism was chronic autoimmune thyroiditis (n = 7), and surgery therapy (n = 4).	Serum
Theodoropoulou A (16)	2009	Greece	Cohort study	7	7	Hyperthyroidism (Graves’ disease)	Serum
Tanda ML (15)	2009	Italy	Cohort study	272	109	The hyperthyroid group consisted of 76 patients, and etiology of the hyperthyroidism was Graves’ disease (n = 46), toxic multinodular goiter (n = 10), and subacute thyroiditis (n = 3) or excess L-T4 TSH-suppressive doses (n = 17). The hypothyroid group consisted of 52 patients and the etiology of the hypothyroidism was chronic autoimmune thyroiditis.144 euthyroid patients with chronic autoimmune thyroiditis.	Plasma
Sawicka B (17)	2010	Poland	Case-control and cohort study	63	15	The hyperthyroid group consisted of 31 patients, and etiology of the hyperthyroidism was Graves’ disease. The hypothyroid group consisted of 32 patients and the etiology of the hypothyroidism was chronic autoimmune thyroiditis.	Serum
Kosowicz J (18)	2011	Poland	Case-control study	49	17	The hyperthyroid group consisted of 24 patients, and etiology of the hyperthyroidism was Graves’ disease. The hypothyroid group consisted of 25 patients and the etiology of surgery therapy.	Plasma
Gurgul E (20)	2012	Poland	Case-control study	62	21	The hyperthyroid group consisted of 22 patients, and etiology of the hyperthyroidism was Graves’ disease. The hypothyroid group consisted of 40 patients and the etiology of the hypothyroidism was Hashimoto’s thyroiditis or after radioiodine treatment (n = 16), and L-thyroxine withdrawal (n = 24).	Plasma
El GS (19)	2012	Egypt	Case-control and cohort study	40	30	Hyperthyroidism (Graves’ disease)	Plasma
Dutta P (6)	2012	India	Case-control and cohort study	27	28	The hyperthyroid group consisted of 27 patients, and etiology of the hyperthyroidism was Graves’ disease (n = 20), and toxic multinodular goiter (n = 7).	Plasma
Ruchala M (24)	2014	Poland	Case-control study	32	16	Hyperthyroidism and hypothyroidism	Plasma
Malandrino N (23)	2014	Rome	Case-control study	25	25	Euthyroid (Hashimoto’s thyroiditis)	Plasma
Biyikli HH (22)	2014	Turkey	Case-control study	48	41	Euthyroid (Hashimoto’s thyroiditis)	Serum
Agbaht K (21)	2014	Turkey	Cohort study	40	40	The hyperthyroid group consisted of 40 patients, and etiology of the hyperthyroidism was Graves’ disease (n = 31), and toxic multinodular goiter (n = 9).	Plasma
Kim KJ (25)	2015	Korea	Case-control study	94	61	The hyperthyroid group consisted of 57 patients, and etiology of the hyperthyroidism was Graves’ disease. The hypothyroid group consisted of 36 patients and the etiology of the hypothyroidism was chronic autoimmune thyroiditis (n = 33), and surgery therapy (n = 3).	Serum
Ucan B (26)	2017	Turkey	Case-control study	54	24	Thyroid carcinoma (papillary thyroid carcinoma)	Plasma
Mele C (27)	2019	Italy	Case-control study	30	20	Thyroid carcinoma (differentiated thyroid carcinoma)	Plasma
Patil A (28)	2022	India	Cohort study	35	35	Hyperthyroidism	Plasma

### Results of meta-analysis

**Figure 2:**
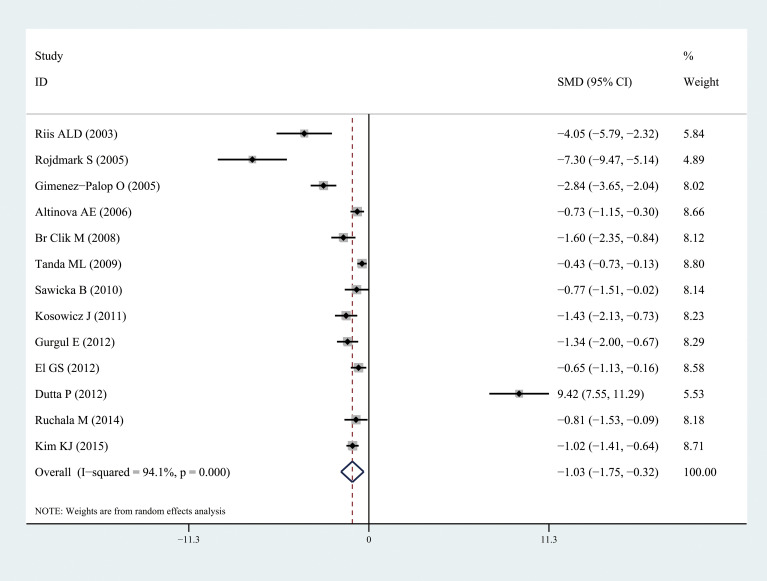
Forest plots of ghrelin level in patients with hyperthyroidism compared to healthy individuals. Diamond represents the pooled SMDs at 95% CI. SMD, standardized mean difference; CI, confidence interval.

**Figure 3:**
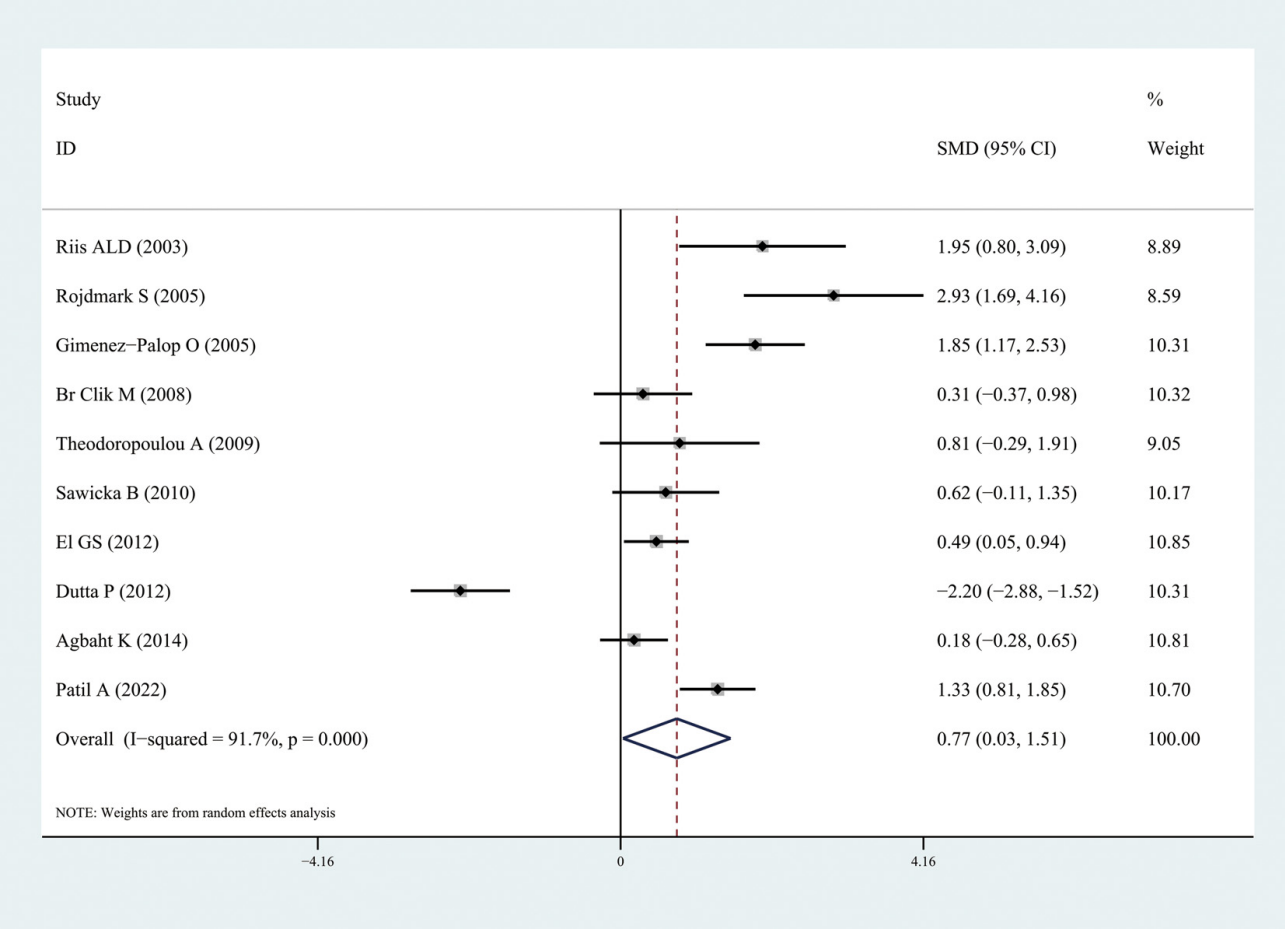
Forest plots of ghrelin level in patients with hyperthyroidism after treatment compared to before. Diamond represents the pooled SMDs at 95% CI. SMD, standardized mean difference; CI, confidence interval.

**Figure 4:**
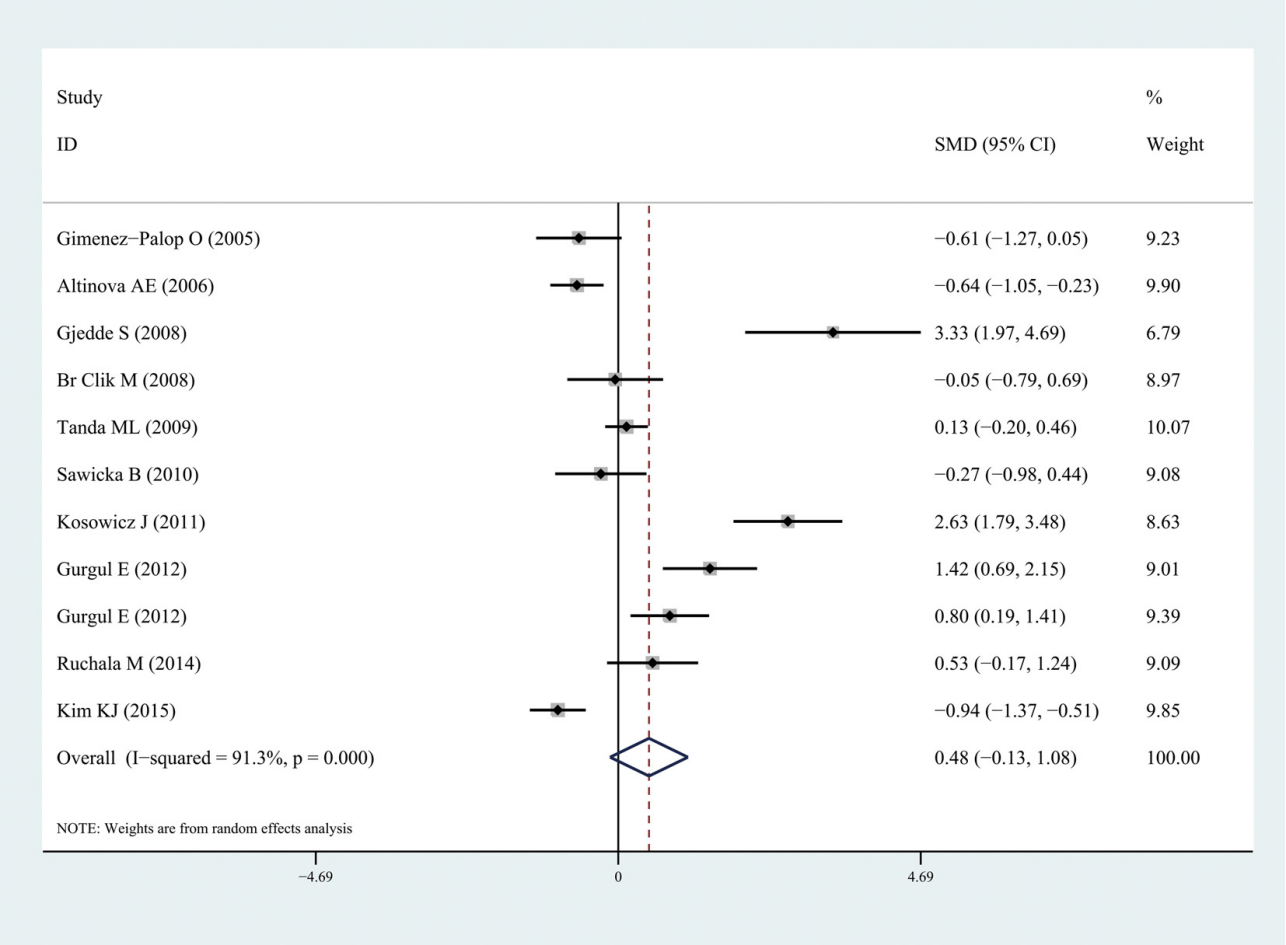
Forest plots of ghrelin level in patients with hypothyroidism compared to healthy individuals. Diamond represents the pooled SMDs at 95% CI. SMD, standardized mean difference; CI, confidence interval.

**Figure 5:**
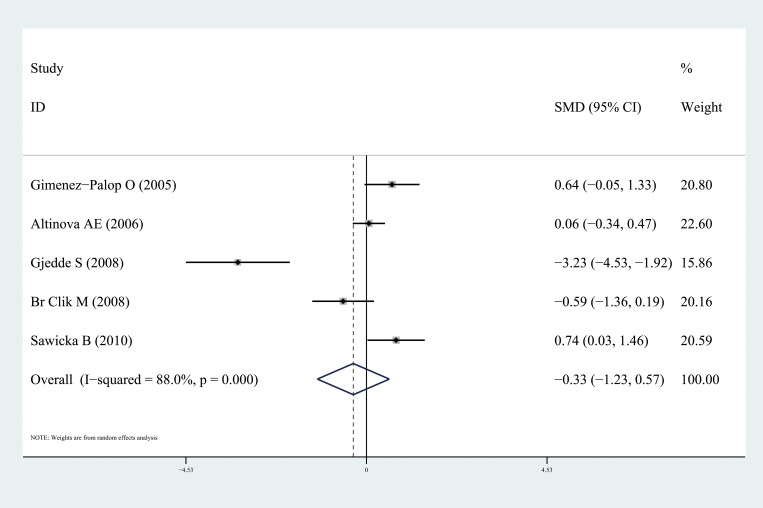
Forest plots of ghrelin level in patients with hypothyroidism after treatment compared to before. Diamond represents the pooled SMDs at 95% CI. SMD, standardized mean difference; CI, confidence interval.

Ghrelin levels were significantly lower in patients with hyperthyroidism than those in healthy individuals (SMD: -1.03, 95% CI [-1.75, 0.32]). Forest plots of the results are presented in **Figure 2**. Ghrelin levels of patients with hyperthyroidism were significantly higher after effective treatment (SMD: 0.77, 95% CI [0.03, 1.51]) (**Figure 3**). Ghrelin levels were not significantly higher in patients with hypothyroidism than in healthy controls (SMD: 0.48, 95% CI [-0.13, 1.08]) (**Figure 4**). And there was also no significant difference of ghrelin levels in patients with hypothyroidism after effective treatment (SMD: -0.33, 95% CI [-1.23, 0.57]) (**Figure 5**). Four studies about euthyroid patients with Hashimoto’s thyroiditis and three studies about thyroid carcinomas, there were both no significant difference of ghrelin levels compared to the healthy controls (SMD: -0.52, 95% CI [-0.82, 1.85] and SMD: 0.36, 95% CI [-1.03, 1.76]).

### Sensitivity analysis and publication bias

To ensure the reliability of the results, the calculations were recalculated by stepwise elimination of ≥ 1 studies and sensitivity analysis using a random-effects model. The results of sensitivity analysis were presented in **Supplementary Figures S1**–**S4**. A thorough and comprehensive database search was performed. Tests for the presence of publication bias in the results of systematic reviews were performed. Egger’s tests were used to assess overall publication bias in the included studies, with no bias observed in any of the included studies.

## Discussion

This systematic review is the first comprehensive evaluation of ghrelin levels in patients with thyroid disease. Twenty-three independent studies were included in this meta-analysis. We concluded that ghrelin levels were significantly lower in patients with hyperthyroidism than in healthy controls, and ghrelin levels of patients with hyperthyroidism were significantly higher after treatment. Ghrelin levels were higher—but not significantly—in patients with hypothyroidism than those in healthy controls.

Ghrelin is an endogenous growth hormone-releasing peptide, so it is a ligand of GHS-R. When combined with pituitary GHS-R, it strongly promotes the release of GH. Ghrelin is mainly synthesized in type X/A cells in the fundus of the stomach, and approximately 60%–70% of circulating ghrelin is derived from the stomach, whereas most of the remainder is derived from the small intestine (2). Ghrelin can promote the secretion of gastric acid, enhance gastric motility, stimulate appetite, increase food intake, increase the absorption of nutrients, especially lipids, promote adipogenesis, affect endocrine function of the pancreas and glucose metabolism, and regulate energy balance (29). Ghrelin production depends on food intake: it increases in the fasting state and decreases after meals; ghrelin levels negatively correlate with body mass: its concentration increases in malnutrition and decreases in obesity. Ghrelin promotes the secretion of adrenocorticotropic hormone, glucocorticoids, prolactin, and other hormones. Raghay et al. detected the distribution of ghrelin in human and rat thyroid tissues using immunohistochemical techniques and found that ghrelin was secreted by thyroid C cells in human and rat thyroid tissues (30). A higher ghrelin were observed in children with higher normal TSH concentration than those in children with lower normal TSH (31).

Increased appetite and weight loss are common among patients with hyperthyroidism. Increased ghrelin secretion can also increase appetite; however, it has been found that ghrelin levels in patients with hyperthyroidism are lower than those in healthy controls, suggesting that hyperthyroidism-induced increase in food intake is not mediated by ghrelin. The same conclusion was reached in animal experiments. Caminos et al. found that the messenger RNA (mRNA) expression of ghrelin in rats with hyperthyroidism was significantly lower than that in the healthy control group (32). Correspondingly, serum ghrelin level was also significantly lower than that in the healthy control group. Most studies have shown that hyperinsulinemia and hyperglycemia inhibit ghrelin secretion. A study by Soriano-Guillen reported that plasma ghrelin levels in 28 obese children who underwent an oral glucose tolerance test were significantly decreased, and when plasma glucose concentration reached its maximum, plasma ghrelin concentration was at its lowest (33). Leonetti et al. found that the hyperinsulinemic state of 35 obese subjects who underwent a hyperinsulinemic euglycemic clamp test exhibited reduced plasma ghrelin levels after continuous insulin infusion (34). Thyroxine promotes intestinal glucose absorption, accelerates the oxidative utilization of glucose and hepatic glycogenolysis, affects insulin secretion and action, and causes insulin resistance by reducing insulin secretion and peripheral insulin sensitivity. Moreover, several clinical studies confirmed that patients with hyperthyroidism have increased Homeostatic Model Assessment for Insulin Resistance (i.e., “HOMA-IR”) indices. Tong et al. confirmed that ghrelin inhibits glucose-induced insulin release and consumption by injecting healthy individuals with exogenous ghrelin (35).

Thyroid hormones have multiple effects on cholesterol levels. They promote cholesterol metabolism and affect cholesterol synthesis and transport. Patients with hyperthyroidism often exhibit clinical manifestations such as decreased blood lipid levels. Because ghrelin function is predicated on binding to lipoproteins, a drop in blood lipid levels can cause a corresponding decrease in ghrelin levels (36). In addition, elevated thyroid hormone levels stimulate the renin-angiotensin-aldosterone system, leading to increased renal blood flow and glomerular filtration rates. This also causes ghrelin to be metabolized at a faster rate, thereby reducing its concentration in the blood. Additionally, it is known that hyperthyroidism is associated with increased activity of the sympathetic nervous system and with abnormalities in the growth hormone/insulin-like growth factor 1 axis, which may affect glucose homeostasis, insulin sensitivity, and ghrelin levels (37). In general, these mechanisms lead to a decrease in blood ghrelin levels in patients with hyperthyroidism, which is a compensatory change in the body. In addition, ghrelin levels have been found to be significantly increased in patients with hyperthyroidism after effective treatment. Therefore, ghrelin levels may also be an effective indicator of hyperthyroidism treatment in the future.

In this study, ghrelin levels in patients with hypothyroidism were higher than those in healthy individuals; however, the difference was not statistically significant. There is a close relationship between hypothyroidism and hyperlipidemia. Thyroid hormones can affect the synthesis and degradation of cholesterol. The synthesis and degradation of lipids in patients with hypothyroidism are decreased, resulting in an increase in blood lipid concentration, which leads to an increase in ghrelin levels. In addition, the rate of metabolism is reduced in patients with hypothyroidism. Increased ghrelin levels may also be a compensatory response to change in appetite and intake caused by decreased thyroid hormone levels. These pathophysiological events may lead to changes in ghrelin levels.

Kanamoto et al. used polymerase chain reaction and immunocytochemical techniques to demonstrate that both the human thyroid follicular carcinoma cell line and the TT cell line of medullary thyroid carcinoma synthesize and secrete ghrelin *in vitro* (38). The TT cell line of medullary thyroid carcinoma synthesized more ghrelin than the TT cell line of thyroid follicular carcinoma. The N-PAP cell line and ARO cell line derived from undifferentiated thyroid carcinoma also produced ghrelin, and the treatment of these cell lines with ghrelin at a concentration of 100 nmol/L to 1 μmol/L exhibited a dose-dependent inhibition of cell proliferation. Because thyroid carcinomas is associated with insulin resistance, ghrelin levels in patients with thyroid carcinomas were analyzed. The results of 3 related studies demonstrated no significant difference in ghrelin levels between patients with thyroid carcinomas and healthy individuals; however, more research is needed to determine the relationship between them.

Hattori et al. first reported the presence of GHS-R in immune cells. Subsequent studies have revealed that immune cells express ghrelin as well as ghrelin receptors. Expression of ghrelin mRNA has been observed in normal human T lymphocytes, B lymphocytes, and neutrophils (39). In some inflammatory diseases, such as inflammatory bowel disease, ankylosing spondylitis, and sepsis, circulating ghrelin levels are significantly increased and are correlated with disease status (40). Several studies have analyzed changes in ghrelin levels in patients with Hashimoto’s thyroiditis with normal thyroid function and found no significant difference from the normal population. However, due to the small number of samples included, it was difficult to draw definitive conclusions. Ghrelin can significantly inhibit the production of inflammatory cytokines by inflammatory cells, such as interleukin (IL)-1, IL-6, and tumor necrosis factor (TNF)-α. IL-1α, interferon-gamma and TNF-α can down-regulate sodium-iodine symporter and affect thyroid hormone synthesis (41). These results suggest that ghrelin plays a role in the pathogenesis of autoimmune thyroid disease and may be used to control the inflammatory response caused by lymphocytes during the treatment of this disease.

The present study had some limitations. First, the methods used to measure ghrelin levels varied among studies. Graves’ disease is the most common cause of hyperthyroidism, whereas Hashimoto’s thyroiditis and thyroid surgery are the most common causes of hypothyroidism. Different causes of hypothyroidism can affect ghrelin levels. Third, studies investigating patients with euthyroid Hashimoto’s thyroiditis, thyroid carcinomas, and ghrelin levels are limited. Therefore, more experimental studies about treatment of thyroid disease by ghrelin is needed. As such, the results of the present meta-analysis should be interpreted with caution.

## Conclusion

This systematic review is the first to evaluate the relationship between ghrelin and thyroid disease. Ghrelin levels were significantly lower in patients with hyperthyroidism. Determining the role of ghrelin in thyroid disease will significantly contribute to understand of symptom or pathomechanism.

## Data Availability

The original contributions presented in the study are included in the article/**Supplementary Material**. Further inquiries can be directed to the corresponding author/s.

## Supplementary material

Supplementary Figure 1 The sensitivity analysis results of ghrelin level in patients with hyperthyroidism compared to healthy individuals.

Supplementary Figure 2 The sensitivity analysis results of ghrelin level in patients with hyperthyroidism after treatment compared to before.

Supplementary Figure 3 The sensitivity analysis results of ghrelin level in patients with hypothyroidism compared to healthy individuals.

Supplementary Figure 4 The sensitivity analysis results of ghrelin level in patients with hypothyroidism after treatment compared to before.
