# Supplementary figures and images for: Relationship between ghrelin and thyroid disease: a meta-analysis
Source: Front Endocrinol (Lausanne). 2025 Feb 28;16:1505085. doi: 10.3389/fendo.2025.1505085 (PMC11906317; doi:10.3389/fendo.2025.1505085)

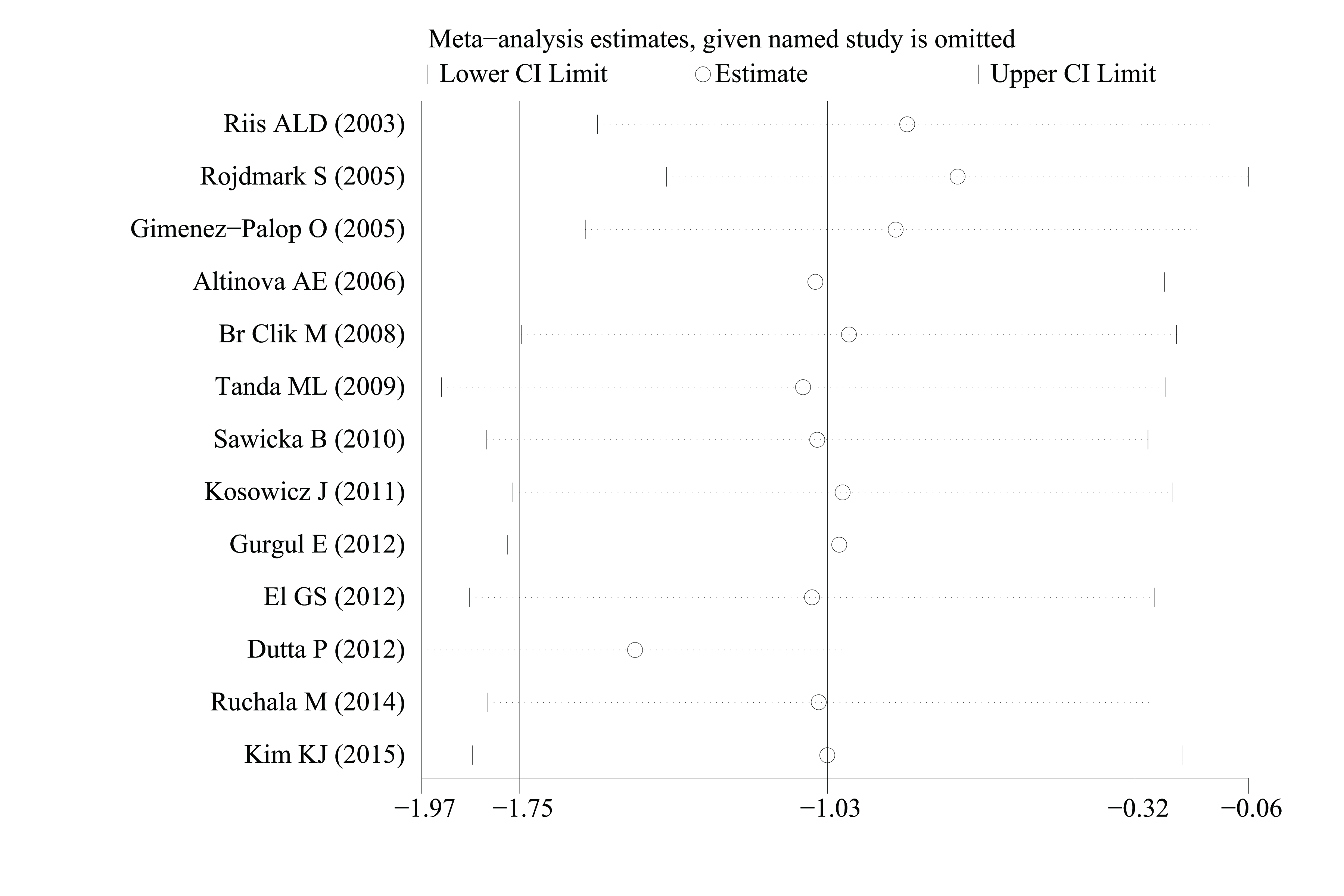

Supplement: Supplementary Figure 1 — The sensitivity analysis results of ghrelin level in patients with hyperthyroidism compared to healthy individuals. [file Image1.tif]

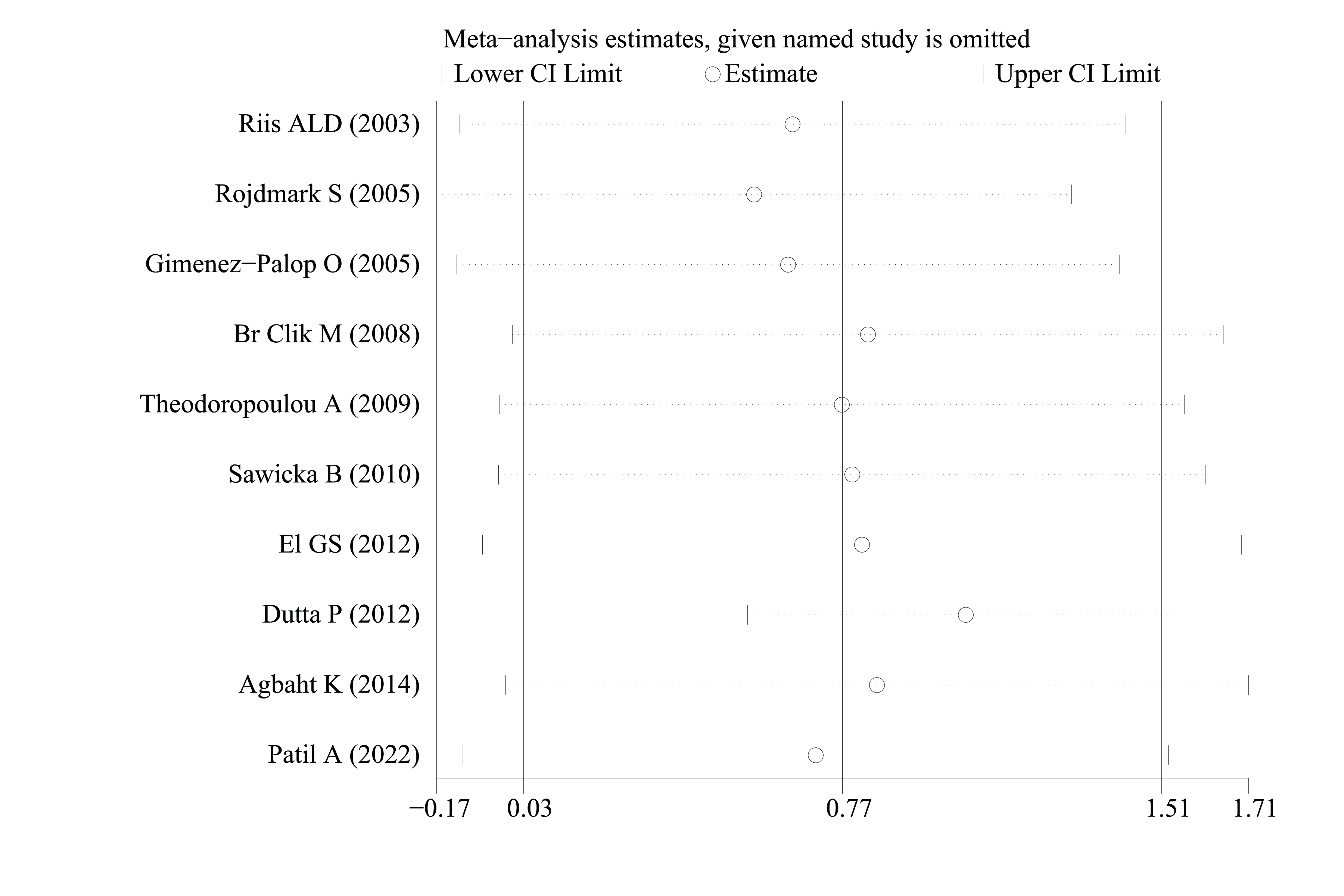

Supplement: Supplementary Figure 2 — The sensitivity analysis results of ghrelin level in patients with hyperthyroidism after treatment compared to before. [file Image2.tif]

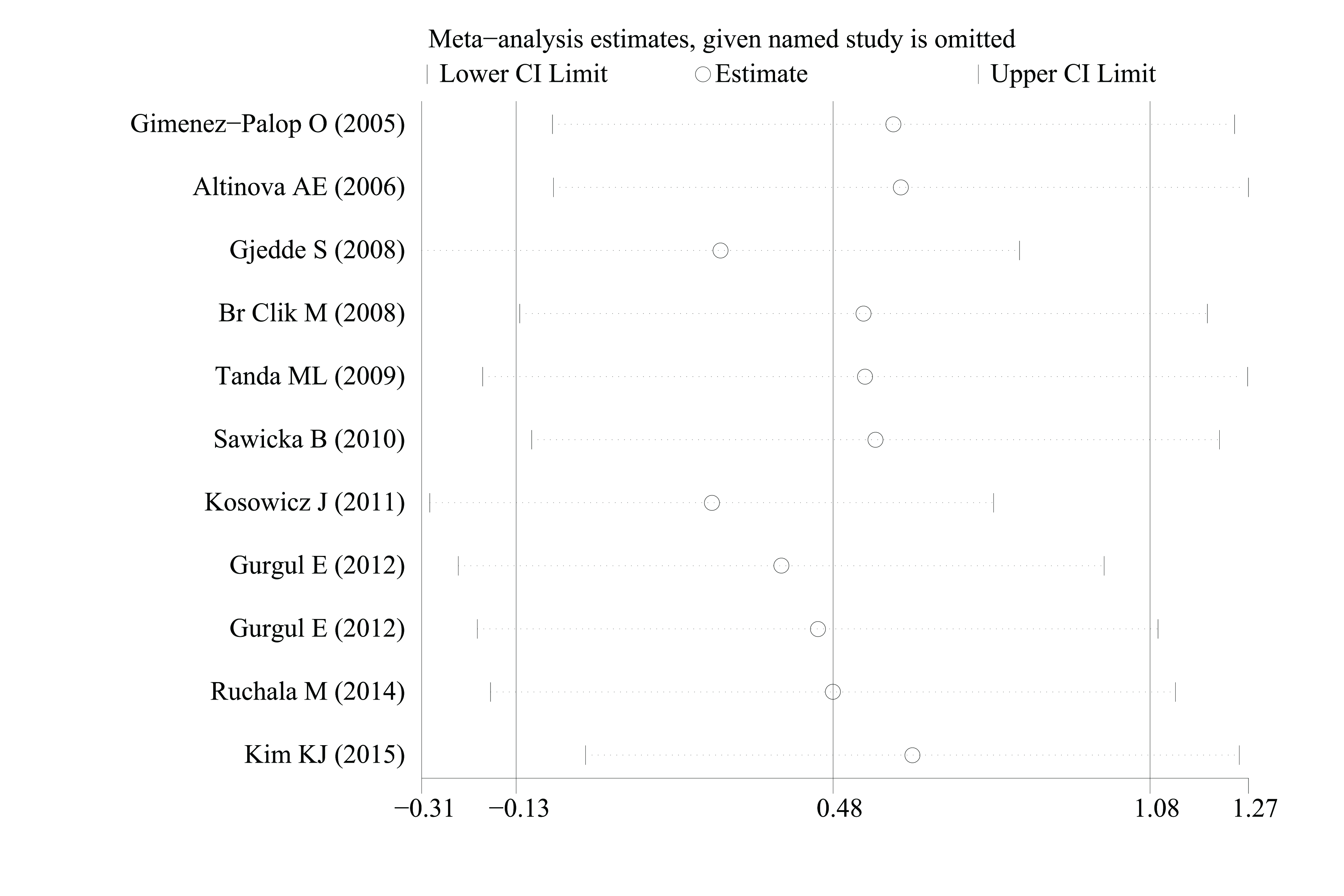

Supplement: Supplementary Figure 3 — The sensitivity analysis results of ghrelin level in patients with hypothyroidism compared to healthy individuals. [file Image3.tif]

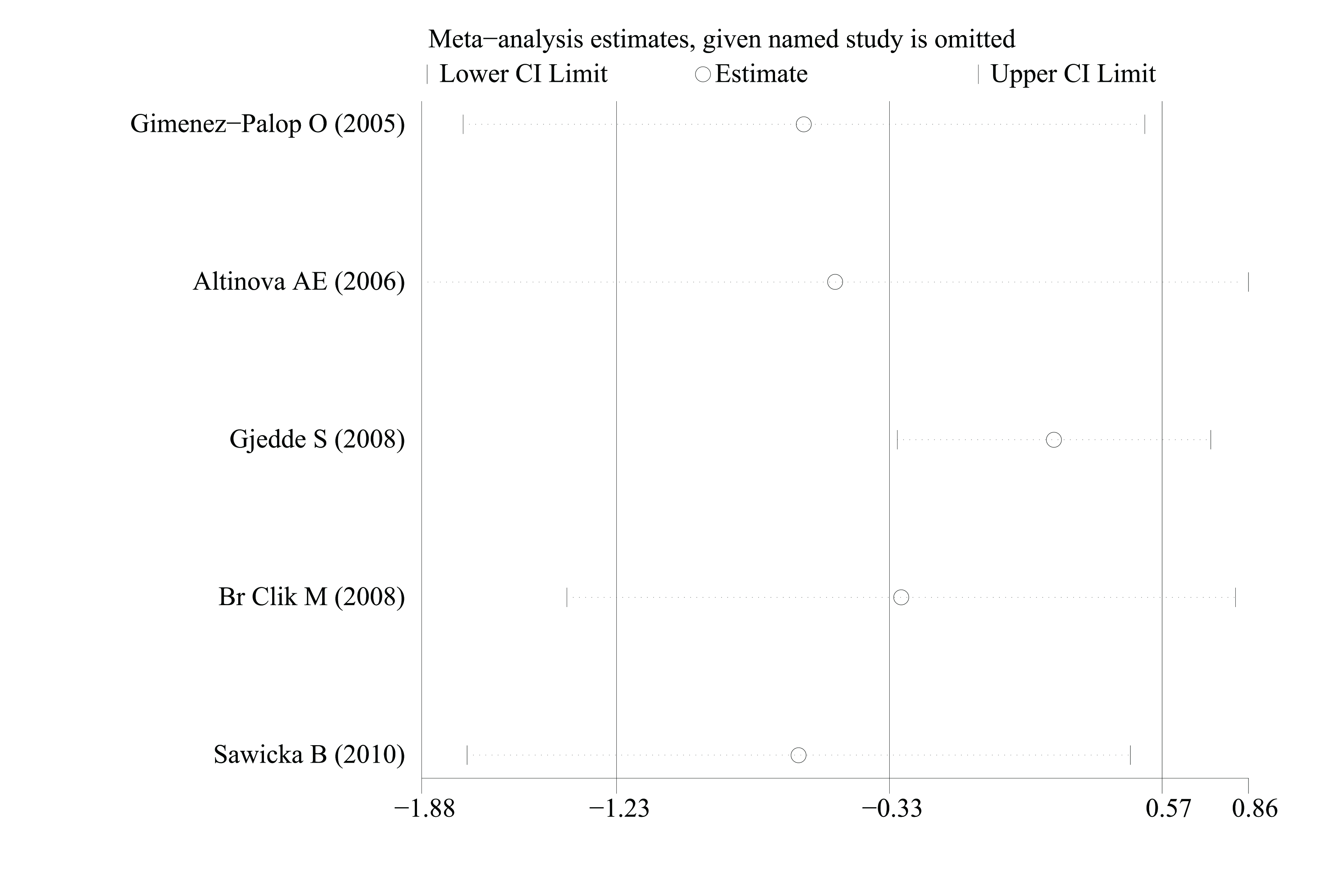

Supplement: Supplementary Figure 4 — The sensitivity analysis results of ghrelin level in patients with hypothyroidism after treatment compared to before. [file Image4.tif]
