# Supplementary material for: Relationship between ghrelin and thyroid disease: a meta-analysis
Source: Front Endocrinol (Lausanne). 2025 Feb 28;16:1505085. doi: 10.3389/fendo.2025.1505085 (PMC11906317; doi:10.3389/fendo.2025.1505085)
Supplement: Supplementary file 6 [file DataSheet2.docx]

Table S2 ROBINS-I Assessment of Study Bias for Included Studies

| **Author** | **Publication Year** | **Confounding** | **Selection of participants into the study** | **Classification of exposures** | **Deviations from intended exposures** | **Missing data** | **Measurement of outcomes** | **Selection of the reported result** | **Risk of bias scores** |
| --- | --- | --- | --- | --- | --- | --- | --- | --- | --- |
| Riis ALD | 2003 | Moderate | Low | Low | Low | Low | Low | Moderate | Moderate |
| Rojdmark S | 2005 | Moderate | Low | Moderate | Low | Low | Low | Moderate | Moderate |
| Morpurgo PS | 2005 | Low | Low | Low | Low | Low | Low | Low | Low |
| Gimenez-Palop O | 2005 | Low | Low | Low | Low | Low | Low | Low | Low |
| Altinova AE(a） | 2006 | Low | Low | Low | Low | Low | Low | Low | Low |
| Altinova AE(b) | 2006 | Moderate | Moderate | Low | Low | Low | Low | Low | Low |
| Gjedde S | 2008 | Moderate | Low | Low | Low | Low | Low | Moderate | Moderate |
| Br Clik M | 2008 | Moderate | Low | Moderate | Low | Low | Low | Moderate | Moderate |
| Theodoropoulou A | 2009 | Low | Low | Low | Low | Low | Low | Low | Low |
| Tanda ML | 2009 | Low | Low | Moderate | Low | Low | Low | Low | Low |
| Sawicka B | 2010 | Low | Low | Low | Low | Low | Low | Low | Low |
| Kosowicz J | 2011 | Moderate | Moderate | Low | Low | Low | Low | Low | Moderate |
| Gurgul E | 2012 | Moderate | Moderate | Low | Low | Low | Low | Low | Moderate |
| El GS | 2012 | Moderate | Low | Low | Low | Low | Low | Moderate | Moderate |
| Dutta P | 2012 | Low | Low | Low | Low | Low | Low | Low | Low |
| Ruchala M | 2014 | Low | Low | Low | Low | Low | Low | Low | Low |
| Malandrino N | 2014 | Low | Moderate | Low | Low | Low | Low | Low | Low |
| Biyikli HH | 2014 | Moderate | Moderate | Low | Low | Low | Low | Low | Moderate |
| Agbaht K | 2014 | Moderate | Low | Low | Low | Low | Low | Moderate | Low |
| Kim KJ | 2015 | Moderate | Low | Moderate | Low | Low | Low | Moderate | Moderate |
| Ucan B | 2017 | Low | Moderate | Low | Low | Low | Low | Low | Low |
| Mele C | 2019 | Low | Low | Low | Low | Low | Low | Low | Low |
| Patil A | 2022 | Moderate | Low | Low | Low | Low | Low | Moderate | Low |
